# Anchoring Vignettes in the Health and Retirement Study: How Do Medical Professionals and Disability Recipients Characterize the Severity of Work Limitations?

**DOI:** 10.1371/journal.pone.0126218

**Published:** 2015-05-12

**Authors:** Frank Heiland, Na Yin

**Affiliations:** 1 School of Public Affairs, Baruch College, City University of New York, New York, New York, United States of America; 2 CUNY Institute for Demographic Research, New York, New York, United States of America; 3 The Graduate Center of CUNY, New York, New York, United States of America; San Francisco, UNITED STATES

## Abstract

**Purpose:**

Recent studies report systematic differences in how individuals categorize the severity of identical health and work limitation vignettes. We investigate how health professionals and disability recipients characterize the severity of work limitations and whether their reporting patterns are robust to demographic, education, and health characteristics. We use the results to illustrate the potential impact of reporting heterogeneity on the distribution of work disability estimated from self-reported categorical health and disability data.

**Method:**

Nationally representative data on anchoring disability vignettes from the 2004 Health and Retirement Study (HRS) are used to investigate how respondents with an occupation background in health and Social Security disability beneficiaries categorize work limitation vignettes. Using pain, cardiovascular health, and depression vignettes, we estimate generalized ordered probit models (*N* = 2,660 individuals or 39,681 person-vignette observations) that allow the severity thresholds to vary by respondent characteristics.

**Results:**

We find that health professionals (excluding nurses) and disability recipients tend to classify identical work limitations as more severe compared to non-health professional non-disabled respondents. For disability recipients, the differences are most pronounced and particularly visible in the tails of the work limitations distribution. For health professionals, we observe smaller differences, affecting primarily the classification of mildly and moderately severe work limitations. The patterns for health professionals (excluding nurses) are robust to demographics, education, and health conditions. The greater likelihood of viewing the vignette person as more severely work limited observed among disability recipients is mostly explained by the fact that these respondents also tend to be in poorer health which itself predicts a more inclusive scale.

**Conclusions:**

Knowledge of reporting scales from health professionals and disabled individuals can benefit researchers in a broad range of applications in health and disability research. They may be useful as reference scales to evaluate disability survey data. Such knowledge may be beneficial when studying disability programs. Given the increasing availability of anchoring vignette data in surveys, this is a promising area for future evaluation research.

## Introduction

Vignette data are becoming increasingly popular in social science research. For health and disability vignettes, respondents are asked to rate, using an ordinal categorical scale, the severity of the health or work limitations of (hypothetical) individuals. A number of recent studies employ anchoring vignette strategies to adjust for reporting heterogeneity in self-reported measures of health and disability [1–9]. These analyses document substantial systematic variation in the way individuals characterize the severity of health conditions or work limitations presented in a vignette. This paper examines how health professionals and disability recipients characterize the severity of work limitations of 15 disability vignettes from the Health and Retirement Study (HRS).

The ratings of health professionals and disability recipients are of interest for a number of reasons. Health professionals are likely to be familiar with a wide range of health limitations and communicating about the severity of a person’s health condition may be part of their job. In turn, the way health professionals characterize the severity levels of individuals in vignettes may serve as an important reference. We also look at respondents who are beneficiaries in the two primary public long-term disability programs in the U.S., the Social Security Disability Insurance and the Supplemental Security Income program. The programs are the main cash transfer programs for disabled workers and their dependents. They provide partial earnings replacement to workers who lose earnings capacity due to severe and long-term disabilities. Social Security disability beneficiaries will have personal experience with health problems and work limitations. They will also have witnessed a rigorous evaluation process determining that their own physical or mental impairments are considered so severe that they are no longer able to perform substantial gainful activities.Detailed discussion about the disability determination process can be found in previous literature [10]. As such, they provide an interesting comparison group to non-disabled individuals as well as health professionals. Furthermore, estimates of reporting scales from important reference groups such as health professionals may be useful to researchers who are interested in studying the sensitivity of their analyses to reporting heterogeneity, for example, in the context of disability benefit application behavior.

Previous research has paid little attention to reporting heterogeneity by occupation or disability status. To date, no study has looked at the vignette responses of health professionals. We identify individuals with the relevant occupational background using measures provided in the confidential data records of the Health and Retirement Study (HRS). We test for heterogeneity in reporting within health professionals by looking at nurses separately, and we explore the role of respondents’ demographics, education, and health. We analyze 15 disability vignettes, five in each of the following three health domains: pain, cardiovascular health, and depression. The categories can be directly linked to the three diagnostic groups (musculoskeletal system and connective tissue, circulatory system, mental disorder) that make up the majority of awards in the Social Security disability programs.

We look at the patterns of reporting heterogeneity in light of potential explanations including exposure effects, empathy bias and justification bias. Sympathy is an emotional response to others’ misfortune. Psychological experiments show that evoking sympathy prompts prosocial behavior. In addition, sympathy could be boosted by reducing the perceived social distance [11–12]. When health professionals rate a given disability vignette, familiarity with the health problem may affect their ability to empathize. As a result, they may be more sympathetic because of their interaction with patients with such problems. On the other hand, their familiarity primarily with more severe cases may result in stricter ratings for the vignette person if the condition of the vignette person is less severe. Their assessment is also likely more accurate about more severe health problems. So we do not have strong theoretical priors regarding the reporting bias for health professionals.

Having health and work limitations themselves may make disabled individuals empathize with the vignette characters who are work limited. Justification bias suggests that respondents may over-report their work limitation to justify their not working [13]. Possible justification bias (justifying their not working and receiving disability benefits) may also make them apply more inclusive scales when they classify a given health limitation. On the other hand, it is possible that disability recipients are stricter and rate a given health problem as less limiting when they compare the vignette to their own disability which is the most severe work limitation (full and permanent disability) according to the program definition of disability. Their experience coping with health limitations may also affect how they perceive disability severity but the direction of the effects is unclear: They could be more empathetic knowing the pain coping with disability. Alternatively, they may gradually adapt to the health limitations well enough to not easily consider a health problem as limiting or insurmountable. Thus we do not have unambiguous predictions regarding the reporting bias for disability recipients.

We find that health professionals (excluding nurses) and disability recipients tend to characterize (identical) work limitations as more serious compared to other respondents. For disability recipients, the differences are most pronounced, particularly in the tails of the work limitations distribution. For health professionals (excluding nurses), we observe smaller differences that primarily affect the classification of mildly and moderately severe work limitations. The patterns for health professionals (excluding nurses) are robust to demographics, education, and health conditions. The differences for disability recipients can be explained in large part by their poor health status.

These findings have broader implications for research relying on self-reported measures of health and disability. We illustrate how the classification scales used by health professionals and disability recipients can be used as reference scales to study the sensitivity of measures of self-reported disability to reporting heterogeneity. We quantify the magnitude of the effect of reporting scales on self-reported disability measures and discuss how it may affect Social Security disability benefit application behavior.

## Analytical Framework

Researchers interested in individuals’ health and work limitations often rely on self-reported categorical measures. Important concerns with self-classifications of the severity of health and work limitations are so-called “scaling” or “anchoring” effects [14]. (Ordinal categorical rating variables have a long tradition in survey research [15]. Five-point Likert scales are among the most commonly used instruments.)Specifically, if the mapping of the true degree of health limitations into the severity categories varies with respondent characteristics, then the self-reported measures are subject to non-random error and conclusions based on these measures will be biased.

**Fig 1:**
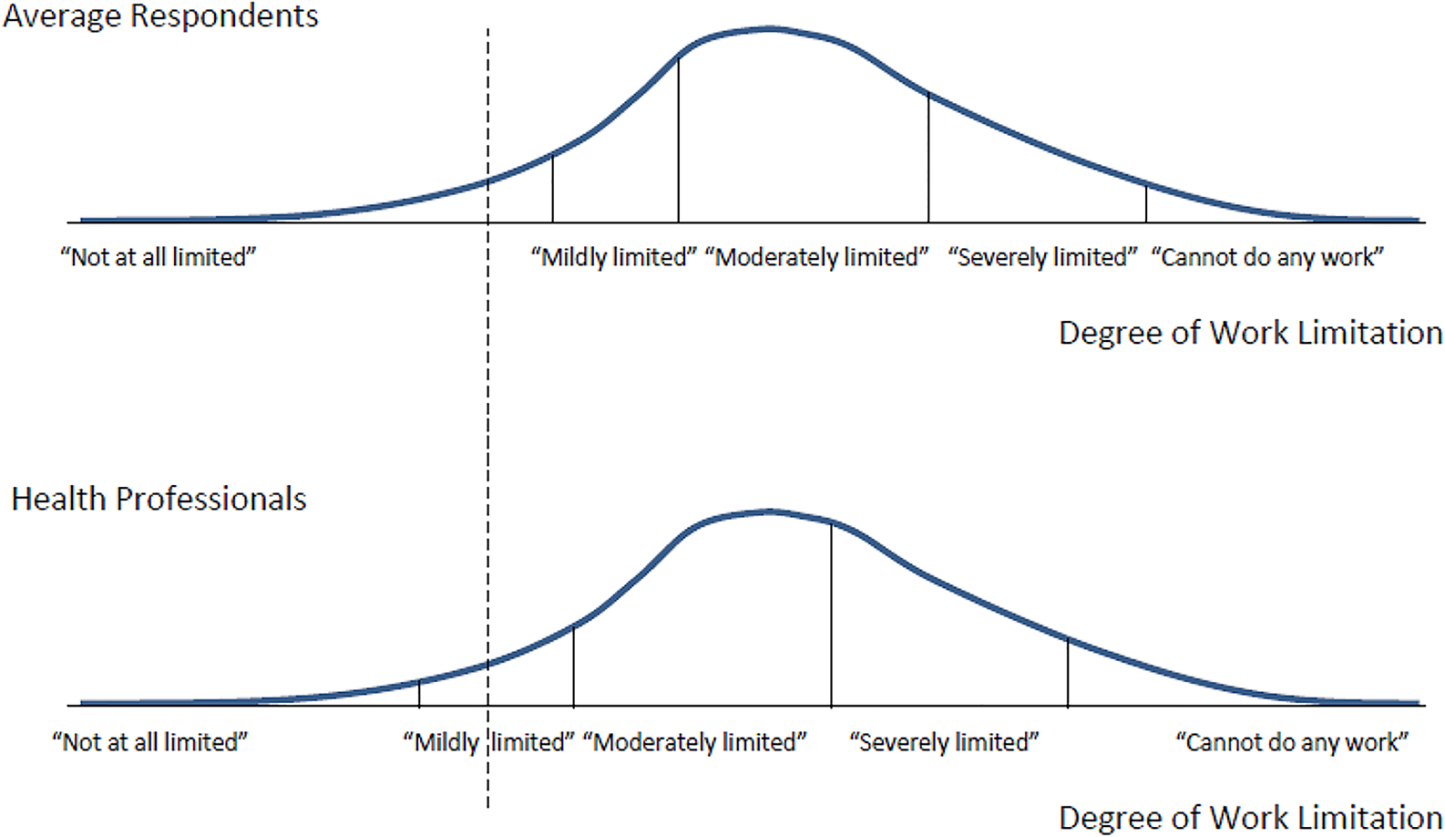
Reporting of Work Limitations across Two Groups in Case of Scale Heterogeneity.

Graphically, this problem can be illustrated, for example, by looking at the differences in the location of the four thresholds (or ‘cut-points’) that respondents use to determine the severity category on a standard five-point response scale. In Fig 1, a person with the same objective degree of work limitations—represented by the dotted vertical line—is classified as not having any work disability by respondents in the top panel (“average respondents” who are neither health professionals nor disability recipients), while respondents from the second group (“health professionals”) would characterize the same person as (mildly) work limited.

Anchoring vignettes have been used to adjust self-reported health and disability measures for differences in classification styles. Disability vignettes ask survey respondents to assess how severely disabled (hypothetical) persons are. Since the vignettes are identical, any variation in the responses across individuals can be attributed to reporting style and may be used to purge individuals’ (own) health assessments of scaling effects. (Previous literature has discussed the anchoring vignette strategy to purge individuals’ own health assessments of scaling effects. We are briefly reviewing this approach in Section V.)

This paper analyzes the differences in the disability classification scales between health professionals, disability recipients, and other “typical” respondents (non-health professionals and non-disability recipients). Health professionals have longer medical training and practice and likely come in contact with a wider range of health problems compared to the non-health-professional and non-disability-recipients respondents. Disability recipients provide an interesting contrast to either group, as they have (severe) work limitations and some first-hand experience with the disability evaluation and award process. We examine whether there are any systematic differences in disability rating styles between those groups, and if so, between those groups we study the robustness of these differences to demographic, education and health characteristics.

Our analytical framework is based on a generalized ordered probit model [16–17] where the cut-points used in classifying the severity of work disability vignettes are allowed to vary with the respondents’ characteristics, *x*_*i*_. We assume a response scale with five categories, consistent with typical vignette data. For each vignette j, there exists a latent true degree of work limitations, *α*_*j*_, that all individuals agree on (net of random error). (This assumption is known as vignette equivalence.) This rules out any association between the degree of disability of the vignette person, $d_{ij}^{v*}$, and the respondent’s characteristics. Consequently, the latent health of each vignette *j* as perceived by individual *i* can be specified as an intercept plus random measurement error:

$$d_{ij}^{v*}=\alpha_{j}+\varepsilon_{ij}^{v},\;\varepsilon_{ij}^{v}∼N\left(0,1\right)$$ (1)

with the normalization *α*_1_ = 0, and $\varepsilon_{ij}^{v}$ independent of each other and of *x*_*i*_.

The respective observed categorical rating $d_{ij}^{v}$ is related to $d_{ij}^{v*}$ through the following mechanism:

$$d_{ij}^{v}=k\;if\;\mu_{i}^{k-1}\leq d_{ij}^{v*}<\mu_{i}^{k},\;k=0,\;1,\ldots ,\;5$$ (2)

where $\mu_{i}^{0}<\mu_{i}^{1}<\mu_{i}^{2}\ldots <\mu_{i}^{5}$, and $\mu_{i}^{0}=-\infty \;and\;\mu_{i}^{5}=+\infty$. The exclusion restriction in (1) permits identification of cut-points as functions of covariates:

$$\mu_{i}^{k}=\gamma_{0}^{k}+x_{i}\gamma^{k}+HP_{i}\beta^{k}+D_{i}\delta^{k},\;k=1,\ldots ,4$$ (3)

where *HP*_*i*_ is a binary variable indicating whether a respondent *i* is health professional. *D*_*i*_ is an indicator of whether a respondent *i* is receiving disability benefits. The effect *β* ^*k*^ measures the differential in response scales between health professionals and non-professionals. The effect *δ* ^*k*^ captures any reporting heterogeneity between disability recipients and non-recipients. As indicated in Eq (3), all coefficients ($\gamma_{0}^{k},\gamma^{k},\beta^{k},\;\text{and}\;\delta^{k}$) can differ across cut-points ($\mu_{i}^{k},k=1,\ldots ,\;4$). In other words, we allow for the possibility that the extent of reporting heterogeneity differs across levels of work limitations.

The categorical scale of work limitation ranges from “not at all limited” to “cannot do any work” and increases in severity. A positive estimate of *β* ^*k*^ (*δ* ^*k*^) would suggest that health professionals (disability recipients) are less inclusive, that is, less likely to rate a given (vignette) person as (more severely) work disabled than non-professionals (non-recipients). Conversely, a negative estimate of *β* ^*k*^ (*δ* ^*k*^) would suggest more inclusive criteria among health professionals (disability recipients) in rating the work limitations vignettes compared to non-professionals (non-recipients).

If health professionals or disability recipients were more accurate at determining disability severity (at the latent level), we would expect less residual variance and less effect of covariates when HP and D are included in the model. Because the residual variance is normalized at 1, we would expect the thresholds to be more spread out in that case, which is consistent with our findings below. (We thank an anonymous referee for pointing this out.) To investigate this further, we have also estimated models with and without HP (health professionals excluding nurses), nurses, and D (disability recipients) dummies. Likelihood-ratio test shows that the HP and D dummies are jointly significant at the 0.01% level, suggesting that including the HP and D dummies significantly improves model fit. Wald test statistics for the null that the coefficients of the other covariates in the threshold equations are jointly equal to zero become smaller but mostly remain significant after we include HP and D dummies in the model. This is also consistent with the idea that health professionals and disability recipients are important predictors of the thresholds and that these groups may provide relatively more accurate assessments.

## Data

We use the 2004 wave of the Health and Retirement Study (HRS), a bi-annual panel with a representative sample of the US population aged over 50 and their spouses. The HRS is sponsored by the National Institute on Aging (grant number NIA U01AG009740) and is conducted by the University of Michigan. Information collected includes physical and mental health, socio-economic status (including measures of labor market status, income, education and wealth), social support, etc. We analyze data from the disability vignette survey, which is based on a subsample of 2004 HRS respondents who completed a face-to-face interview and a drop-off questionnaire consisting of a series of work disability vignettes. Throughout our analysis, we apply the sampling weights available in the HRS for vignette respondents. The weights are the product of three factors: a) the HRS respondent-level weight for the 2004 wave, b) a face-to-face interview adjustment factor, and c) a non-response adjustment factor for the specific drop-off questionnaire (see http://hrsonline.isr.umich.edu/modules/meta/tracker/desc/LBWeights2004_Description_public.pdf).

The anchoring vignettes deal with typical health problems in the domains of pain, depression, and cardio-vascular health. The categories can be directly linked to the three diagnostic groups—musculoskeletal system and connective tissue (32.8%), mental disorders (14.1%), and circulatory system (15.1%)—that made up 62% of all Social Security disability awards to Americans age 50 and above in 2004 (SSA, 2004, Table 38) [18].

In each of the three domains five distinct vignettes are used to describe the condition of a hypothetical person. An example of a vignette from the “pain” domain is the following (Pain Vignette No. 5, see the vignettes questionnaire in S1 Appendix): *Jane has pain in her back and legs*, *and the pain is present almost all the time*. *It gets worse while she is working*. *Although medication helps*, *she feels uncomfortable when moving around*, *holding and lifting things at work*. For each vignette, the respondent is asked: “How much is [Jane] limited in the kind or amount of work she could do?” 1—“Not at all limited”; 2—“Mildly limited”; 3—“Moderately limited”; 4—“Severely limited”; 5—“Cannot do any work”. (We list all 15 disability vignettes in the S1 Appendix.)

Health professionals are identified from a restricted HRS data file on industry and occupation. The file contains detailed respondent-level industry and occupation information following the 1980 Census occupational classification system for all interview years. Based on this information, we identify 258 sample members who have ever worked in one of the following health-related occupations (we report the frequency in the analytical sample in parentheses): health diagnosing occupation, i.e. physicians (5.40% of 258 health professionals), registered nurses (19.27%) and other health assessment and treating occupations (4.25%), therapists (1.54%), medical scientist (1.16%), medical science or health specialties teachers (1.16%), health technologists and technicians (18.91%), and health service occupations (48.30%). (There are 29 individuals in the data who are both health professionals and disability recipients.)

Using these health professional groups, we create two binary indicators: A dummy for registered nurses and a dummy for individuals with an occupational background in health profession other than nurses. The vignette responses by physicians and registered nurses who have clinic training and experience are particularly interesting. However, due to the limited sample size of physicians, we would not emphasize our estimation results from them.

We contrast the reporting patterns of health professionals to those of disability recipients and other non-health professional non-disabled respondents. We define as disability beneficiaries those individuals who receive benefits from Social Security Disability Insurance or Supplemental Security Income program, the two primary public long-term disability programs in the United States for workers and their eligible dependents.

To explore explanations for differences in reporting scales across these groups, we employ a set of individual-level variables including basic demographics (age, sex, race/ethnicity, and marital status), educational attainment (“less than high school”, “high school”, “some college”, “college or more”), a set of binary health indicators (high blood pressure or hypertension; diabetes or high blood sugar; cancer or a malignant tumor or any kind except skin cancer; chronic lung disease except asthma such as chronic bronchitis or emphysema; heart attack, coronary heart disease, angina, congestive heart failure, or other heart problems; arthritis or rheumatism; emotional, nervous, or psychiatric problems; and whether the person is obese), and difficulties in Activities of Daily Living (ADLs).

Table 1 provides descriptive statistics for an array of demographic and health variables for our analytical sample and for the sub-samples of health professionals and disability recipients. Vignette ratings by health domain are also summarized in the table. As shown in the table, there are 2,660 individuals in our main sample, contributing 39,681 observations (person-vignettes) in total with a loss of 0.05 percent of observations due to missing information on vignette ratings. (In the regression analysis, we include indicators for missing data on education and health.)We have 258 health professionals and 193 disability recipients, contributing 3,856 and 2,883 observations, respectively. Looking at the simple (unconditional) sample means across health domains, we see that disability recipients rate the vignette’s work limitations as more severe than other groups.

**Table 1:** Sample Descriptives.

	Whole Sample			Health Professionals			Disability Recipients		
	Mean	SD		Mean	SD		Mean	SD	
Male	0.42	0.49		0.14	0.35		0.45	0.50	
Age	63.28	6.99		62.84	7.04		61.81	6.15	
Age 50–55	0.20	0.40		0.21	0.41		0.20	0.40	
Age 56–60	0.14	0.35		0.16	0.36		0.20	0.40	
Age 61–65	0.22	0.41		0.21	0.41		0.31	0.46	
Age 66–70	0.25	0.44		0.27	0.44		0.22	0.42	
Age 70+	0.18	0.39		0.15	0.35		0.07	0.26	
Married	0.73	0.45		0.66	0.48		0.60	0.49	
*Race/ethnicity*									
non-Hispnic White	0.76	0.43		0.75	0.43		0.54	0.50	
non-Hispnic Black	0.15	0.36		0.19	0.39		0.36	0.48	
non-Hispnic other	0.02	0.14		0.01	0.11		0.04	0.19	
Hispanic	0.07	0.26		0.05	0.23		0.07	0.25	
*Education*									
less than high school	0.20	0.40		0.13	0.34		0.36	0.48	
high school	0.35	0.48		0.25	0.43		0.35	0.48	
some college	0.22	0.41		0.32	0.47		0.16	0.37	
college+	0.22	0.42		0.28	0.45		0.13	0.34	
*Health conditions*									
high blood pressure or hypertension	0.51	0.50		0.52	0.50		0.70	0.46	
diabetes or high blood sugar	0.18	0.38		0.18	0.38		0.36	0.48	
cancer or a malignant tumor	0.11	0.32		0.13	0.33		0.13	0.34	
chronic lung disease	0.09	0.28		0.09	0.29		0.23	0.42	
heart problems	0.18	0.39		0.18	0.39		0.38	0.49	
arthritis or rheumatism	0.54	0.50		0.62	0.48		0.77	0.42	
cesd score	1.27	1.92		1.40	1.97		2.98	2.54	
Obesity	0.31	0.46		0.36	0.48		0.44	0.50	
*#Observations (N)*	39,681			3,856			2,883		
*#Individuals*	2,660			258			193		
Vignette rating: affect	2.31	1.22	*N = 13*,*218*	2.33	1.25	*N = 1*,*288*	2.38	1.26	*N = 961*
Vignette rating: pain	3.10	1.22	*N = 13*,*239*	3.10	1.23	*N = 1*,*287*	3.33	1.21	*N = 962*
Vignette rating: cvd	2.90	1.31	*N = 13*,*224*	2.89	1.32	*N = 1*,*281*	3.10	1.35	*N = 960*

*Notes*: Vignette ratings are coded as follows: 1—“Not at all limited”, 2—“Mildly limited”, 3—“Moderately limited”, 4—“Severely limited”, and 5—“Cannot do any work”.

## Main Results

Table 2 shows the estimated coefficients for health professionals excluding nurses, nurses, and individuals receiving disability benefits based on the threshold Eq (3). The four panels in the table correspond to four different model specifications. Model 1 only includes the health professional dummies and the indicator for individuals receiving disability benefits (plus a constant term). Model 2 controls for basic demographic factors (gender, age, race/ethnicity, and marital status). Model 3 controls for educational attainment in addition to basic demographics. Model 4 controls for a set of health indicators (high blood pressure, diabetes, cancer, chronic lung disease, heart problems, arthritis, mental health problems, obesity, and ADLs) in addition to the demographic and education variables.

**Table 2:** Estimation Results in Generalized Ordered Probit Model of Respondents' Rating of Vignettes' Work Limitation.

	**Model 1**			
	mu1	mu2	mu3	mu4
	Not at all limited = > mildly limited	mildly limited = >moderately limited	moderately limited = > severely limited	severely limited = > cannot do any work
Health Professionals (Excl. Nurses)	-0.116	-0.164**	-0.158**	-0.113
	(0.084)	(0.070)	(0.071)	(0.079)
Health Professionals: Nurses	0.022	0.124	0.205***	0.319***
	(0.095)	(0.081)	(0.072)	(0.097)
Disability Recipients	-0.188**	-0.184***	-0.191***	-0.435***
	(0.079)	(0.064)	(0.062)	(0.099)
Demographics	No			
Education	No			
Health Conditions	No			
*N*	39,681			
*Log likelihood*	-45,502			
	**Model 2**			
	mu1	mu2	mu3	mu4
Health Professionals (Excl. Nurses)	-0.144*	-0.167***	-0.140**	-0.027
	(0.084)	(0.068)	(0.069)	(0.081)
Health Professionals: Nurses	-0.023	0.064	0.131*	0.210**
	(0.095)	(0.080)	(0.072)	(0.096)
Disability Recipients	-0.176**	-0.146**	-0.130**	-0.301***
	(0.082)	(0.065)	(0.061)	(0.092)
Demographics	Yes			
Education	No			
Health Conditions	No			
*N*	39,681			
*Log likelihood*	-45,056			
	**Model 3**			
	mu1	mu2	mu3	mu4
Health Professionals (Excl. Nurses)	-0.146*	-0.175***	-0.154**	-0.046
	(0.083)	(0.068)	(0.069)	(0.083)
Health Professionals: Nurses	-0.022	0.050	0.104	0.137
	(0.096)	(0.081)	(0.073)	(0.100)
Disability Recipients	-0.185**	-0.136**	-0.111*	-0.265***
	(0.082)	(0.065)	(0.061)	(0.093)
Demographics	Yes			
Education	Yes			
Health Conditions	No			
*N*	39,681			
*Log likelihood*	-44,959			
	**Model 4**			
	mu1	mu2	mu3	mu4
Health Professionals (Excl. Nurses)	-0.139*	-0.175***	-0.154**	-0.057
	(0.084)	(0.069)	(0.071)	(0.087)
Health Professionals: Nurses	-0.021	0.055	0.112	0.127
	(0.096)	(0.081)	(0.074)	(0.101)
Disability Recipients	-0.106	-0.060	-0.013	-0.092
	(0.082)	(0.066)	(0.068)	(0.115)
Demographics	Yes			
Education	Yes			
Health Conditions	Yes			
*N*	39,681			
*Log likelihood*	-44,760			

*Notes*: Demographic variables include gender, age, race and marital status. Health conditions include: high blood pressure or hypertension; diabetes or high blood sugar; cancer or a malignant tumor or any kind except skin cancer; chronic lung disease except asthma such as chronic bronchitis or emphysema; heart attack, coronary heart disease, angina, congestive heart failure, or other heart problems; arthritis or rheumatism; emotional, nervous, or psychiatric problems; obesity; and number of Activities of Daily Living difficulties. The estimation also includes indicators for missing data on education and health. Standard errors are clustered at individual levels shown in parenthesis. ***, ** and * indicate statistical significance at p<.01, p<.05 and p<.10 levels, respectively, and two-tailed tests.

Horizontally, the results are presented by cut-point on the severity scale from “Mildly limited vs. Not at all limited” (μ_1_) to “Cannot do any work vs. Severely limited” (μ_4_). A positive coefficient associated with health professionals or disability recipients implies a shift up (right) in the cut-point, suggesting that, on average, individuals from the corresponding group characterize the work limitations presented in the vignette as less severe. We report standard errors that are clustered at the individual level, allowing for potential correlation in an individual’s vignette responses.

Looking at the results for disability recipients in Model 1, we find statistically significant negative coefficients for all cut-points. At any severity level, disability recipients tend to characterize the vignette person’s limitations as more work limiting than do non-recipients. This pattern is more pronounced at the upper tail of the distribution, suggesting that disability recipients, compared to non-recipients, are more likely to think that the vignette person cannot do any work.

Turning to the results for health professionals in Model 1, we find that the coefficients for the health professional indicator (excl. nurses) are negative throughout and largest and statistically significant at the 5% significance level or better for the cut-points μ_2_ and μ_3_. The results suggest that this group, on average, applies a more inclusive scale when rating the severity of mild and moderate work limitations compared to non-health professionals. Specifically, they are more likely to consider the condition described in the vignette as mildly or moderately work limiting than non-disabled non-health professionals.

Model 1 also points to some diversity in the assessments within health professionals as there is evidence that the nurses apply a stricter scale than the average person and other health professionals. For nurses, we observe positive coefficients throughout that become larger and more statistically significant across the disability severity distribution.

Models 2 and 3 explore the robustness of the above patterns to demographic variables. Model 2 shows that the estimates for health professionals (excl. nurses) change little when gender, age, and marital status are controlled for. The coefficients associated with disability recipients are smaller by up to one-third in Model 2 compared to Model 1, but remain statistically significant at conventional levels. The coefficients associated with being a nurse decrease significantly and maintain only marginal significance. Looking at the independent effects of race/ethnicity (see Table A in S2 Appendix), we see that non-Hispanic whites apply a stricter scale than other racial/ethnic groups, especially at higher severity levels. This suggests that the more lenient reporting scale found for disability recipients in Model 1 is in part a reflection of the smaller concentration of non-Hispanic whites among the disabled group. Similarly, the stricter scale observed for individuals who worked as nurses in Models 1 appears to be partly driven by the fact that health professionals in that group are more likely to be non-Hispanic whites.

Models 3 and 4 explore education and health as potential mediating variables in addition to the demographic measures in Model 2. Accounting for educational attainment in Model 3, the pattern is found to be similar to Model 2 for disability recipients and health professionals (excl. nurses) and the coefficients change little. However, the coefficients for the nurses decline further relative to Models 1 and 2 and are no longer significant.

As many disability recipients are of lower socio-economic status (see Table 1), we might expect substantial changes in the coefficients on recipiency after education is controlled for. We do not see that, which suggests that socio-economic status is not the main driver for the observed classification differences. The findings for nurses suggest that the stricter scale when assessing more severe cases observed for this group may be attributed in part to their educational attainment. Nurses are most likely to have education beyond high school and the latter is a strong predictor of applying stricter thresholds μ_3_ and μ_4_ (see Table A in S2 Appendix).

As shown in Model 4, the pattern associated with being a health professional (excl. nurses) is robust to controls for individuals’ health (in addition to controls for education). However, the coefficients associated with receiving disability are smaller in absolute terms in Model 4 compared to Model 3 and are no longer significant at conventional levels. This suggests, not surprisingly, that the greater likelihood of viewing the vignette person as more severely work limited observed among disability recipients is partly explained by the fact that these respondents also tend to be in poorer health, which itself predicts a more inclusive scale at the tails of the disability distribution (see Table A in S2 Appendix).

**Fig 2:**
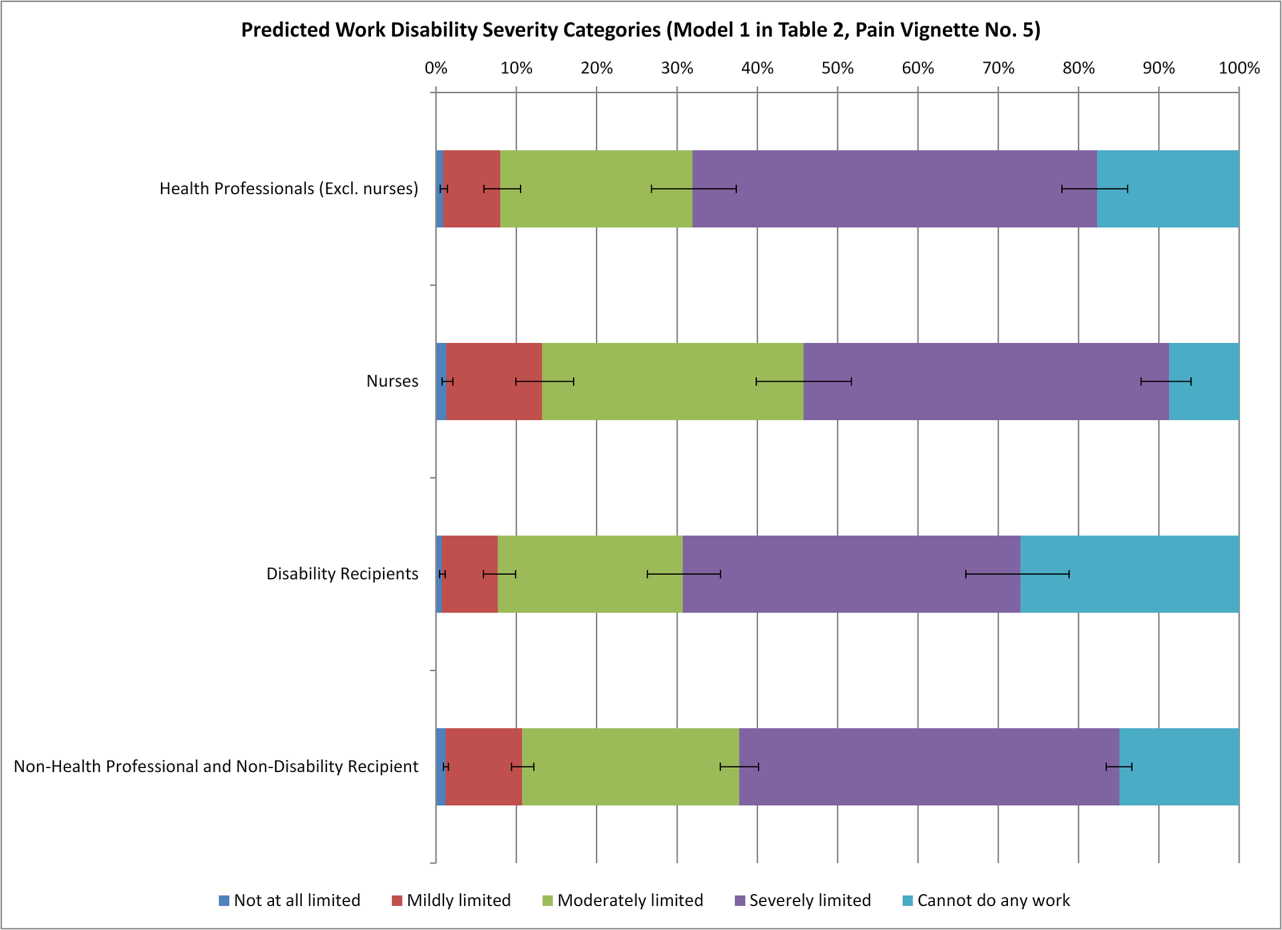
Predicted Distribution of Work Disability Severity Categories (Model 1 in Table 2, Pain Vignette No. 5).

To illustrate the magnitudes of the estimated relationships, we can graph the predicted distribution of the severity categories for a given vignette (j). This distribution is obtained by applying the classification scale of a given type of respondent (e.g., health professionals) to the distribution of the latent level of work disability associated with that vignette. (As shown in Eq (1), the distribution of the latent disability level associated with vignette j across respondents follows a normal distribution centered around $\hat{\alpha_{j}}$, the estimate of the true latent health level of vignette j, with a standard deviation that is normalized to 1.) Fig 2 shows the predicted distribution of severity categories for health professionals (excl. nurses), nurses, disability recipients, and other respondents based on Model 1 (Table 2) for pain vignette No. 5.We include 95% confidence intervals based on the estimated cut-points.

As discussed above in Model 1, disability recipients, on average, apply the least strict classification scale. Consistent with that, Fig 2 shows that there is a 27% chance that disability recipients report the person in pain vignette No. 5 as extremely work disabled. For average respondents (non-disabled non-health professionals) this probability is only 15%. The distribution of categories “not at all limited”, “mildly limited” and “moderately limited” is similar between health professionals (excl. nurses) and disability recipients. However, the former are less likely to rate the vignette as extremely disabled (18%). Lastly, Fig 2 confirms that nurses tend to apply the strictest scale, resulting in greatest likelihoods of reporting “mildly limited” (12%) and “moderately limited” (33%) across these groups.

## Applications

The response scales inferred from vignette classifications of health professionals or disability beneficiaries in the previous section can be useful to researchers relying on self-reported measures of health and disability. In this section, we conduct simulations to illustrate the potential magnitude of the effect of reporting heterogeneity in estimating the distribution of disability severity from self-reported survey data.

**Fig 3:**
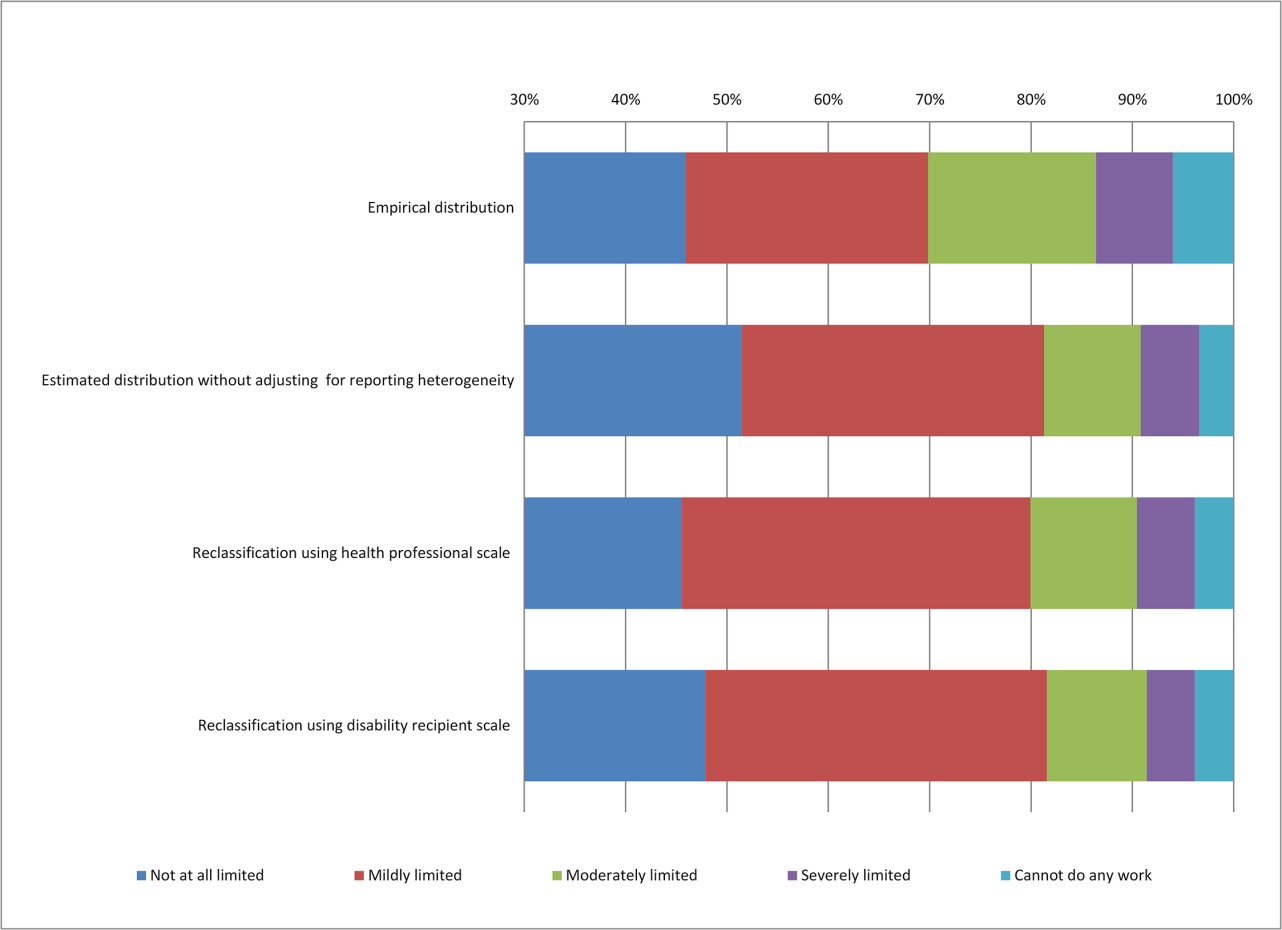
Distribution of Work Limitations (Application). “Empirical distribution” refers to the distribution of self-reported work limitations among HRS respondents who have participated in the vignette questionnaire. “Estimated distribution without adjusting for reporting heterogeneity” refers to the distribution of work limitations estimated using the HOPIT procedure but without adjusting reporting heterogeneity, which is similar to an ordered probit model. “Reclassification using health professional scale” and “Reclassification using disability recipient scale” refers to the distribution of work limitations adjusted for reporting heterogeneity in accordance with the estimated scales (based on Model 4 in Table 2) for health professionals (excluding nurses) and disability recipients.

The top graph in Fig 3 shows the empirical (unadjusted) distribution of disability severity categories among older Americans in the HRS. The other three graphs represent the distribution of severity categories predicted from a Hierarchical Ordered Probit (HOPIT) model [17]. The HOPIT procedures jointly model the processes of reporting style and own health (work limitations). Anchoring vignette data are used to identify the reporting style component, akin to the cut-point equations in (3). The cut-points link individuals’ latent health to the observed severity categories. Similar to a traditional ordered probit model, the second component of the HOPIT defines the latent level of self-reported own health. However, the HOPIT imposes the estimated person-specific response scales in the own health process, permitting separate identification of health and reporting effects. (Identification in the anchoring vignette approach relies on two assumptions: *vignette equivalence* (described in Section II) and *response consistency*. The latter requires that individuals use the same response scales when classifying the vignette health and their own health. The evidence is mixed on whether or not the two assumptions hold. The tests are usually very demanding and rely on alternative assumptions, limited objective measures, and selective health domains [8, 19–23].)

The second graph in Fig 3 represents a predicted distribution of disability severity categories using HOPIT procedures without any correction for reporting heterogeneity (similar to an ordered probit model). In the latent own health equation, we include the same set of regressors as in Model 4 (demographics, education and health variables). The adjusted disability severity distribution is much more concentrated at no limitations (51.5%) and mild limitations (29.8%) compared to the empirical distribution (46.0% and 23.9%). The other severity categories—those above “mild limitations”—become much less common, once demographic, education and health differences across respondents are accounted for.

The limitation of the HOPIT approach is that the reference scale is arbitrary as it is the group represented by the omitted categories in Eq (3). Applying any reference scale of interest, we can reclassify all responses and make them consistent with that scale. We illustrate the impact of standardization with a reference scale in Fig 3. The bottom two graphs show the predicted distribution of disability categories after applying a HOPIT correction for self-reporting heterogeneity *and* reclassifying all responses in accordance with the scales of health professionals (excl. nurses) and disability recipients (based on Model 4 in Table 2, predicted using respondents’ observed characteristics). The predicted distributions are less concentrated at the category “not at all work limited,” consistent with our finding of greater inclusiveness (in the left tail) of health professionals and disability recipients.

Previous studies suggest that a dichotomous question in household surveys on whether the respondent is work limited or not is an important predictor of her decision to apply for disability benefits [10]. As Kapteyn et al. (2007:466) point out, for such a binary measure, “… being softer on those with a minor condition is much more important than being harder on those with a serious work limitation.” This suggests that the location of the first cut-off point on the five-point work limitations severity scale—the threshold that defines cases with no work limitations—could be most critical when mapping the severity of work limitations onto a binary disability scale.

We can also use our results (Model 4, Table 2) to illustrate the impact of the response scale on standard measures of self-reported work disability. If older Americans were to classify their work limitations on the same scale as health professionals (excl. nurses) (or disability beneficiaries), the proportion reporting work limitations (of any severity level) would increase by 5.9 (3.6) percentage points.

Using a representative sample of older Americans in the HRS, we estimate that being work limited increases the likelihood of applying for disability benefits by about 3.7 percentage points. We predict that the disability application rate of the median person in that generation would go up by about 0.22 (0.13) percentage points, if they applied the vignette classification of health professionals (or disability beneficiary) estimated above. This back-of-the-envelope estimate is consistent with earlier evidence of a sizeable population that does not report any work limitations and does not receive Social Security disability benefits but would be medically eligible [24].

The illustration above of the potential impact of reporting heterogeneity clearly shows that researchers should exercise caution when using (unadjusted) self-reported measures to study the effect of health or disability on behaviors and outcomes.

## Discussion and Conclusions

Self-reported categorical general health and work disability measures are widely used in social science research, but the reliability of such measures in capturing differences in true health and disability status in the population has been questioned [13, 25–28]. An important concern is that different groups of the population use different scales when classifying the severity of their health and work limitations. Evidence from the study of health and disability vignette data confirms the presence of substantial heterogeneity in classification scales [4–7].

In this paper, we examine how health professionals and disability recipients rate the severity of work disability vignettes in the Health and Retirement Study (HRS). We find that most individuals with an occupational background in health professions and current Social Security disability beneficiaries tend to classify identical work limitations as more serious compared to non-disabled non-health professional respondents. For disability recipients the differences are most pronounced but the differences are largely explained by their poor health. The findings seem to cast doubt on the so-called justification hypothesis (disability recipients over-reporting the severity of their disability to justify their not working). For health professionals, we observe different reporting patterns between nurses and other health professionals. Nurses tend to be less inclusive when classifying severe and extreme work limitations but the patterns become less obvious when we control for education. Other health professionals seem to apply more inclusive rating styles, affecting primarily mildly and moderately severe work limitations. The patterns are robust to controls for health and education.

The results seem to suggest that the empathy hypothesis dominates over the other hypotheses that we discussed in the introduction section. Health professionals (excluding nurses) and disability recipients are likely more able to empathize with health limited individuals and on average apply a more inclusive scale in classifying disability severity. Health professionals may be a self-selected group of individuals who care more about the wellbeing of others than the average person. They may become increasingly sympathetic through their contact with health impaired individuals. Social Security disability benefit recipients will have first-hand experience with health and work limitations. As a result, they may be more empathetic than the respondents who are neither disability recipients nor health professionals. The fact that we find robust evidence of health professionals (excl. nurses) applying a more inclusive scale is consistent with the empathy explanation.

We need to take caution while interpreting the results. Although health professionals have received relevant training and experience, they may not be as familiar with the impact of health conditions on work functioning, an aspect that may be important in the disability assessment [29]. Disability awardees have a unique perspective as they will have gone through an extensive evaluation process in which Social Security caseworkers establish that their health conditions are sufficiently severe in the context of their work potential, drawing on medical and occupational expertise as needed (e.g., evaluations by physicians defined by Social Security regulation as “acceptable medical sources”). However, disability recipients may only be knowledgeable in the health domain related to their diagnosed condition, as evidenced by the fact that their more inclusive scales is mainly explained by their own health conditions and functional limitations. Moreover, among individuals with a certain condition, the ones for whom this is a limitation are more likely to apply for disability benefits. Consequently, disability recipients may constitute a population that is biased toward perceiving a condition as more limiting.

Knowledge of reporting scales from health professionals and disabled individuals can benefit researchers in a broad range of applications in health and disability research. They may be useful as reference scales to evaluate disability survey data. Such knowledge may be beneficial when studying disability programs. For example, we can compare the disability evaluations by medical professionals (one “gate keeper” in disability programs), disabled individuals, and other groups to perhaps better understand the health effects on disability applications and receipts. Given the increasing availability of anchoring vignette data in surveys, this is a promising area for future evaluation research.

## Supporting Information

S1 Appendix Data Appendix. (DOCX)

S2 Appendix Table A. (DOCX)

S1 Dataset (XLS)

S2 Dataset (XLS)

S3 Dataset STATA Code 1. (PDF)

S4 Dataset STATA Code 2. (PDF)

S5 Dataset STATA Code 3. (PDF)
