# Supplementary material for: Anchoring Vignettes in the Health and Retirement Study: How Do Medical Professionals and Disability Recipients Characterize the Severity of Work Limitations?
Source: PLoS One. 2015 May 12;10(5):e0126218. doi: 10.1371/journal.pone.0126218 (PMC4428751; doi:10.1371/journal.pone.0126218)
Supplement: S1 Appendix — (DOCX) [file pone.0126218.s001.docx]

**Data Appendix: HRS Vignette questionnaires**

**Depression (“Affect”) Vignettes:**

1. Mary generally enjoys her work. She gets depressed every three weeks for a day or two and loses interest in what she usually enjoys, but is able to carry on with her day-to-day activities on the job.

2. Ann enjoys work very much. She feels that she is doing a very good job and is optimistic about the future.

3. Nancy has mood swings on the job. When she gets depressed, everything she does at work is an effort for her and she no longer enjoys her usual activities at work. These mood swings are not predictable and occur two or three times during a month.

4. Barb feels worried all the time. She gets depressed once a week at work for a couple of days in a row, thinking about what could go wrong and that her boss will disapprove of her condition. But she is able to come out of this mood if she concentrates on something else.

5. Kathleen feels depressed most of the time. She weeps frequently at work and feels hopeless about the future. She feels that she has become a burden on her co-workers and that she would be better dead.

**Pain Vignettes:**

1. Sue occasionally feels back pain at work, but this has not happened for the last several months now. If she feels back pain, it typically lasts only for a few days.

2. Carol suffers from back pain that causes stiffness in her back especially at work but is relieved with low doses of medication. She does not have any pains other than this generalized discomfort.

3. Ruth has almost constant pain in her back and this sometimes prevents her from doing her work.

4. Linda has back pain that makes changes in body position while she is working very uncomfortable. She is unable to stand or sit for more than half an hour. Medicines decrease the pain a little, but it is there all the time and interferes with her ability to carry out even day-to-day tasks at work.

5. Jane has pain in her back and legs, and the pain is present almost all the time. It gets worse while she is working. Although medication helps, she feels uncomfortable when moving around, holding and lifting things at work.

**Cardiovascular Health (“CVD”) Vignettes:**

1. Karen is very active and fit. She takes aerobic classes 3 times a week.

2. Lori has had heart problems in the past and she has been told to watch her cholesterol level. Sometimes if she feels stressed at work she feels pain in her chest and occasionally in her arms.

3. Beth’s family has a history of heart problems. Her father died of a heart attack when Beth was still very young. The doctors have told Beth that she is at severe risk of having a serious heart attack herself and that she should avoid strenuous physical activity or stress. She sometimes feels severe pain in her chest and arms, and suffers from dizziness, fainting, sweating, nausea or shortness of breath.

4. Diane has been diagnosed with high blood pressure. Her blood pressure goes up quickly if she feels under stress. Diane does not exercise much and is overweight. Life can sometimes be hectic for her. She does not get along with her boss very well.

5. Judy has undergone triple bypass heart surgery. She is a heavy smoker and still experiences severe chest pain sometimes.
