# Supplementary material for: Anchoring Vignettes in the Health and Retirement Study: How Do Medical Professionals and Disability Recipients Characterize the Severity of Work Limitations?
Source: PLoS One. 2015 May 12;10(5):e0126218. doi: 10.1371/journal.pone.0126218 (PMC4428751; doi:10.1371/journal.pone.0126218)
Supplement: S2 Appendix — (DOCX) [file pone.0126218.s002.docx]

**Appendix Table A: Generalized Ordered Probit Model of Respondents' Rating of Vignettes' Work Limitation (Model 4)**

|  | mu1 |  | mu2 |  | mu3 |  | mu4 |  |
| --- | --- | --- | --- | --- | --- | --- | --- | --- |
|  | Not at all limited => mildly limited |  | mildly limited =>moderately limited |  | moderately limited => severely limited |  | severely limited => cannot do any work |  |
| Health Professionals (Excl. Nurses) | -0.139 | * | -0.175 | *** | -0.154 | ** | -0.057 |  |
|  | (0.084) |  | (0.069) |  | (0.070) |  | (0.087) |  |
| Health Professionals: Nurses | -0.021 |  | 0.055 |  | 0.112 |  | 0.127 |  |
|  | (0.096) |  | (0.081) |  | (0.074) |  | (0.101) |  |
| Disability Recipients | -0.106 |  | -0.060 |  | -0.013 |  | -0.092 |  |
|  | (0.082) |  | (0.066) |  | (0.068) |  | (0.115) |  |
| *Demographics* |  |  |  |  |  |  |  |  |
| Male | -0.140 | *** | -0.106 | *** | -0.099 | *** | -0.041 |  |
|  | (0.048) |  | (0.037) |  | (0.035) |  | (0.050) |  |
| Age 56-60 (Ref: Age 55-55) | -0.065 |  | -0.052 |  | -0.045 |  | -0.059 |  |
|  | (0.068) |  | (0.053) |  | (0.053) |  | (0.077) |  |
| Age 61-65 | -0.074 |  | -0.038 |  | -0.006 |  | 0.017 |  |
|  | (0.065) |  | (0.050) |  | (0.048) |  | (0.067) |  |
| Age 66-70 | -0.071 |  | -0.079 | * | -0.074 |  | -0.156 | ** |
|  | (0.062) |  | (0.048) |  | (0.048) |  | (0.072) |  |
| Age 70+ | -0.105 |  | -0.153 | *** | -0.102 | ** | -0.077 |  |
|  | (0.074) |  | (0.054) |  | (0.050) |  | (0.069) |  |
| Married | -0.037 |  | -0.031 |  | 0.011 |  | 0.065 |  |
|  | (0.052) |  | (0.042) |  | (0.039) |  | (0.057) |  |
| *Race/ethnicity* (Ref: non-Hispanic White) |  |  |  |  |  |  |  |  |
| Non-Hispnic Black | -0.038 |  | -0.246 | *** | -0.320 | *** | -0.571 | *** |
|  | (0.077) |  | (0.059) |  | (0.050) |  | (0.079) |  |
| Non-Hispnic Other | -0.130 |  | -0.296 | * | -0.246 | * | -0.313 | * |
|  | (0.159) |  | (0.153) |  | (0.137) |  | (0.175) |  |
| Hispanic | -0.043 |  | -0.028 |  | -0.171 | ** | -0.308 | *** |
|  | (0.098) |  | (0.078) |  | (0.072) |  | (0.085) |  |
| *Education* |  |  |  |  |  |  |  |  |
| Less than high school | 0.133 | * | -0.003 |  | -0.008 |  | -0.089 |  |
|  | (0.071) |  | (0.055) |  | (0.053) |  | (0.072) |  |
| Some college | 0.044 |  | 0.032 |  | 0.057 |  | 0.121 | * |
|  | (0.059) |  | (0.046) |  | (0.045) |  | (0.064) |  |
| College+ | 0.020 |  | 0.062 |  | 0.120 | *** | 0.237 | *** |
|  | (0.056) |  | (0.045) |  | (0.045) |  | (0.062) |  |
| *Health conditions* |  |  |  |  |  |  |  |  |
| High blood pressure or hypertension | -0.046 |  | -0.056 |  | -0.076 | ** | 0.014 |  |
|  | (0.047) |  | (0.036) |  | (0.035) |  | (0.055) |  |
| Diabetes or high blood sugar | -0.021 |  | -0.063 |  | -0.068 |  | -0.064 |  |
|  | (0.063) |  | (0.047) |  | (0.044) |  | (0.072) |  |
| Cancer or a malignant tumor | 0.123 | * | 0.084 |  | 0.062 |  | 0.125 | * |
|  | (0.077) |  | (0.062) |  | (0.056) |  | (0.077) |  |
| Chronic lung disease | 0.100 |  | 0.019 |  | 0.035 |  | 0.059 |  |
|  | (0.080) |  | (0.072) |  | (0.065) |  | (0.085) |  |
| Heart problems | -0.125 | ** | -0.046 |  | -0.040 |  | 0.004 |  |
|  | (0.051) |  | (0.040) |  | (0.044) |  | (0.065) |  |
| Arthritis or rheumatism | -0.001 |  | 0.033 |  | 0.047 |  | 0.075 |  |
|  | (0.047) |  | (0.037) |  | (0.036) |  | (0.054) |  |
| CESD Score | -0.025 | ** | -0.019 | ** | -0.029 | *** | -0.043 | *** |
|  | (0.011) |  | (0.009) |  | (0.010) |  | (0.015) |  |
| Obesity | 0.025 |  | 0.008 |  | -0.037 |  | -0.118 | ** |
|  | (0.051) |  | (0.040) |  | (0.038) |  | (0.052) |  |
| ADLs | -0.016 |  | -0.025 |  | -0.038 |  | -0.132 | *** |
|  | (0.037) |  | (0.031) |  | (0.030) |  | (0.047) |  |
| Constant | -0.033 |  | 0.997 | *** | 1.915 | *** | 3.247 | *** |
|  | (0.077) |  | (0.063) |  | (0.063) |  | (0.087) |  |
| *No. Observations* | *39,681* |  |  |  |  |  |  |  |
| *Log-likelihood* | *-44,760* |  |  |  |  |  |  |  |

*Notes:* The estimation includes indicators for missing data on education and health. Standard errors that are clustered at individual levels are shown in parenthesis***, ** and * indicate statistical significance at p<.01, p<.05 and p<.10 levels, respectively, and two-tailed tests.
