# Supplementary material for: Anchoring Vignettes in the Health and Retirement Study: How Do Medical Professionals and Disability Recipients Characterize the Severity of Work Limitations?
Source: PLoS One. 2015 May 12;10(5):e0126218. doi: 10.1371/journal.pone.0126218 (PMC4428751; doi:10.1371/journal.pone.0126218)
Supplement: S3 Dataset — (PDF) [file pone.0126218.s005.pdf]

```
* This is a STATA do.file and needs to run in STATA.

clear all
set more off


use "S2_Dataset1.dta", clear


* Table 1
su
su if HP_nonnurse==1 | HP_nurse==1
su if DR==1

tabstat vig, by(domain) stat(n mean sd)
tabstat vig if (HP_nonnurse==1 | HP_nurse==1), by(domain) stat(n mean sd)
tabstat vig if DR==1, by(domain) stat(n mean sd)



* Table 2
global xvars1 "HP_nonnurse HP_nurse DR"
global xvars2 "male age5660 age6165 age6670 age70pl nonhispblack nonhispothr hispan married HP_nonnurse HP_nurse DR"
global xvars3 "male age5660 age6165 age6670 age70pl nonhispblack nonhispothr hispan married HP_nonnurse HP_nurse DR lths ltcollege college edumiss"
global xvars4 "male age5660 age6165 age6670 age70pl nonhispblack nonhispothr hispan married HP_nonnurse HP_nurse DR lths ltcollege college edumiss hibpe diabe cancre lung hearte arthre cesd obese adla hlthmiss cesdmiss obesemiss"
run "gop.do"

* Model 1
ml model lf gop (xb: vigdum*, nocons)                          ///
  (mu1: $xvars1) (mu2: $xvars1) (mu3: $xvars1) (mu4: $xvars1) [aw=weight], cluster(hhidpn)
ml search
ml maximize

* Model 2
ml model lf gop (xb: vigdum*, nocons)                          ///
  (mu1: $xvars2) (mu2: $xvars2) (mu3: $xvars2) (mu4: $xvars2) [aw=weight], cluster(hhidpn)
ml search
ml maximize

* Model 3
ml model lf gop (xb: vigdum*, nocons)                          ///
  (mu1: $xvars3) (mu2: $xvars3) (mu3: $xvars3) (mu4: $xvars3) [aw=weight], cluster(hhidpn)
ml search
ml maximize

* Model 4
ml model lf gop (xb: vigdum*, nocons)                          ///
  (mu1: $xvars4) (mu2: $xvars4) (mu3: $xvars4) (mu4: $xvars4) [aw=weight], cluster(hhidpn)
ml search
ml maximize



* Figure 2
use "S2_Dataset2.dta", clear
//Pain Vignette No.5 is the omitted vignette

ml model lf gop (xb: vigdum*, nocons)                          ///
  (mu1: $xvars1) (mu2: $xvars1) (mu3: $xvars1) (mu4: $xvars1) [aw=weight], cluster(hhidpn)
ml search
ml maximize

* Predict the cut-points for subgroups:

* HP excl. nurses
lincom [mu1]_cons+[mu1]HP_nonnurse
```

```
lincom [mu2]_cons+[mu2]HP_nonnurse
lincom [mu3]_cons+[mu3]HP_nonnurse
lincom [mu4]_cons+[mu4]HP_nonnurse

* Nurses
lincom [mu1]_cons+[mu1]HP_nurse
lincom [mu2]_cons+[mu2]HP_nurse
lincom [mu3]_cons+[mu3]HP_nurse
lincom [mu4]_cons+[mu4]HP_nurse

* DR
lincom [mu1]_cons+[mu1]DR
lincom [mu2]_cons+[mu2]DR
lincom [mu3]_cons+[mu3]DR
lincom [mu4]_cons+[mu4]DR

* Non-HP and non-DR
lincom [mu1]_cons
lincom [mu2]_cons
lincom [mu3]_cons
lincom [mu4]_cons



* Figure 3
use "S2_Dataset1.dta", clear
drop vigdum* domain
reshape wide
run "hopit15.do"

* Figure3: Graph2 “Estimated distribution without adjusting for reporting heterogeneity”
ml model lf hopit15 (xb: $xvars4) (sig:) (vig2:) (vig3:) (vig4:) (vig5:) (vig6:) (vig7:)
(vig8:) (vig9:) (vig10:) (vig11:) (vig12:) (vig13:) (vig14:) (vig15:) ///
(mu1:) (mu2:) (mu3:) (mu4:) [aw=weight] if y<., cluster(hhidpn)
ml search
ml maximize

predict y_pr0 if y<.
predict mu1, equation(mu1)
predict mu2, equation(mu2)
predict mu3, equation(mu3)
predict mu4, equation(mu4)

gen y_hat0 =1 if (y_pr0<=mu1)
replace y_hat0 =2 if (y_pr0>mu1) & (y_pr0<=mu2)
replace y_hat0 =3 if (y_pr0>mu2) & (y_pr0<=mu3)
replace y_hat0 =4 if (y_pr0>mu3) & (y_pr0<=mu4)
replace y_hat0 =5 if (y_pr0>mu4)
replace y_hat0 =. if y_pr0>=.

tab y_hat0


* Figure 3: Adjusting for reporting styles
* Graph3 “Reclassification using health professional scale” and Graph4 “Reclassification
using disability recipient scale”

ml model lf hopit15 (xb: $xvars4) (sig:) (vig2:) (vig3:) (vig4:) (vig5:) (vig6:) (vig7:)
(vig8:) (vig9:) (vig10:) (vig11:) (vig12:) (vig13:) (vig14:) (vig15:) ///
(mu1: $xvars4) (mu2: $xvars4) (mu3: $xvars4) (mu4: $xvars4) [aw=weight] if y<.,
cluster(hhidpn)
ml search
ml maximize
predict y_pr if y<.

gen mu1_b = _b[mu1:_cons]+_b[mu1:male]*male+_b[mu1:age5660]*age5660+_b[mu1:age6165]*age6165+
_b[mu1:age6670]*age6670+_b[mu1:age70pl]*age70pl+_b[mu1:nonhispblack]*nonhispblack ///
+_b[mu1:nonhispothr]*nonhispothr+_b[mu1:hispan]*hispan+_b[mu1:married]*married ///
+_b[mu1:lths]*lths+_b[mu1:ltcollege]*ltcollege+_b[mu1:college]*college ///
+_b[mu1:hibpe]*hibpe+_b[mu1:diabe]*diabe+_b[mu1:cancre]*cancre+_b[mu1:lunge]*lunge+_b[mu1:
```

```
hearte]*hearte+_b[mu1:arthre]*arthre ///
+_b[mu1:adla]*adla+_b[mu1:cesdm]*cesdm+_b[mu1:obese]*obese+_b[mu1:DR]*DR

gen mu2_b = _b[mu2:_cons]+_b[mu2:male]*male+_b[mu2:age5660]*age5660+_b[mu2:age6165]*age6165+
_b[mu2:age6670]*age6670+_b[mu2:age70pl]*age70pl+_b[mu2:nonhispblack]*nonhispblack ///
+_b[mu2:nonhispothr]*nonhispothr+_b[mu2:hispan]*hispan+_b[mu2:married]*married ///
+_b[mu2:lths]*lths+_b[mu2:ltcollege]*ltcollege+_b[mu2:college]*college ///
+_b[mu2:hibpe]*hibpe+_b[mu2:diabe]*diabe+_b[mu2:cancre]*cancre+_b[mu2:lunge]*lunge+_b[mu2:
hearte]*hearte+_b[mu2:arthre]*arthre ///
+_b[mu2:adla]*adla+_b[mu2:cesdm]*cesdm+_b[mu2:obese]*obese+_b[mu2:DR]*DR

gen mu3_b = _b[mu3:_cons]+_b[mu3:male]*male+_b[mu3:age5660]*age5660+_b[mu3:age6165]*age6165+
_b[mu3:age6670]*age6670+_b[mu3:age70pl]*age70pl+_b[mu3:nonhispblack]*nonhispblack ///
+_b[mu3:nonhispothr]*nonhispothr+_b[mu3:hispan]*hispan+_b[mu3:married]*married ///
+_b[mu3:lths]*lths+_b[mu3:ltcollege]*ltcollege+_b[mu3:college]*college ///
+_b[mu3:hibpe]*hibpe+_b[mu3:diabe]*diabe+_b[mu3:cancre]*cancre+_b[mu3:lunge]*lunge+_b[mu3:
hearte]*hearte+_b[mu3:arthre]*arthre ///
+_b[mu3:adla]*adla+_b[mu3:cesdm]*cesdm+_b[mu3:obese]*obese+_b[mu3:DR]*DR

gen mu4_b = _b[mu4:_cons]+_b[mu4:male]*male+_b[mu4:age5660]*age5660+_b[mu4:age6165]*age6165+
_b[mu4:age6670]*age6670+_b[mu4:age70pl]*age70pl+_b[mu4:nonhispblack]*nonhispblack ///
+_b[mu4:nonhispothr]*nonhispothr+_b[mu4:hispan]*hispan+_b[mu4:married]*married ///
+_b[mu4:lths]*lths+_b[mu4:ltcollege]*ltcollege+_b[mu4:college]*college ///
+_b[mu4:hibpe]*hibpe+_b[mu4:diabe]*diabe+_b[mu4:cancre]*cancre+_b[mu4:lunge]*lunge+_b[mu4:
hearte]*hearte+_b[mu4:arthre]*arthre ///
+_b[mu4:adla]*adla+_b[mu4:cesdm]*cesdm+_b[mu4:obese]*obese+_b[mu4:DR]*DR

* Cutpoints for HP (excl. nurses)
gen mu1hp = mu1_b+_b[mu1:HP_nonnurse]*1
gen mu2hp = mu2_b+_b[mu2:HP_nonnurse]*1
gen mu3hp = mu3_b+_b[mu3:HP_nonnurse]*1
gen mu4hp = mu4_b+_b[mu4:HP_nonnurse]*1

* Cutpoints for DR
gen mu1dr = mu1_b-_b[mu1:DR]*DR+_b[mu1:DR]*1
gen mu2dr = mu2_b-_b[mu2:DR]*DR+_b[mu2:DR]*1
gen mu3dr = mu3_b-_b[mu3:DR]*DR+_b[mu3:DR]*1
gen mu4dr = mu4_b-_b[mu4:DR]*DR+_b[mu4:DR]*1


gen y_hathp = 1 if (y_pr<=mu1hp)
replace y_hathp =2 if (y_pr>mu1hp) & (y_pr<=mu2hp)
replace y_hathp =3 if (y_pr>mu2hp) & (y_pr<=mu3hp)
replace y_hathp =4 if (y_pr>mu3hp) & (y_pr<=mu4hp)
replace y_hathp =5 if (y_pr>mu4hp)
replace y_hathp =. if y_pr>=.

tab y_hathp

gen y_hatdr = 1 if (y_pr<=mu1dr)
replace y_hatdr =2 if (y_pr>mu1dr) & (y_pr<=mu2dr)
replace y_hatdr =3 if (y_pr>mu2dr) & (y_pr<=mu3dr)
replace y_hatdr =4 if (y_pr>mu3dr) & (y_pr<=mu4dr)
replace y_hatdr =5 if (y_pr>mu4dr)
replace y_hatdr =. if y_pr>=.

tab y_hatdr
```
