# Supplementary material for: Anchoring Vignettes in the Health and Retirement Study: How Do Medical Professionals and Disability Recipients Characterize the Severity of Work Limitations?
Source: PLoS One. 2015 May 12;10(5):e0126218. doi: 10.1371/journal.pone.0126218 (PMC4428751; doi:10.1371/journal.pone.0126218)
Supplement: S4 Dataset — (PDF) [file pone.0126218.s006.pdf]

```
* This is a STATA do.file and needs to run in STATA.
* The code was adapted from Jones, A., Rice, N., Bago d'Uva, T., & Balia, S. (2007). Applied health economics. London, UK: Routledge.

cap program drop gop
program define gop
    args lnf b m1 m2 m3 m4
    tempvar p1 p2 p3 p4 p5

   quietly {
      gen double `p1'=0
      gen double `p2'=0
      gen double `p3'=0
      gen double `p4'=0
      gen double `p5'=0

   replace `p1' = normal(`m1'-`b')
   replace `p2' = normal(`m2'-`b') - normal(`m1'-`b')
   replace `p3' = normal(`m3'-`b') - normal(`m2'-`b')
   replace `p4' = normal(`m4'-`b') - normal(`m3'-`b')
   replace `p5' = 1 - normal(`m4'-`b')

   replace `lnf' = (vig==1)*ln(`p1')+(vig==2)*ln(`p2')+(vig==3)*ln(`p3')+(vig==4)*ln(`p4')+(vig==5)*ln(`p5')
}
end
```
