# Supplementary material for: Anchoring Vignettes in the Health and Retirement Study: How Do Medical Professionals and Disability Recipients Characterize the Severity of Work Limitations?
Source: PLoS One. 2015 May 12;10(5):e0126218. doi: 10.1371/journal.pone.0126218 (PMC4428751; doi:10.1371/journal.pone.0126218)
Supplement: S5 Dataset — (PDF) [file pone.0126218.s007.pdf]

```
* This is a STATA do.file and needs to run in STATA.
* The code was adapted from Jones, A., Rice, N., Bago d'Uva, T., & Balia, S. (2007). Applied health economics. London, UK: Routledge.

#delimit ;
cap program drop hopit15;
program define hopit15;
args lnf b s
         b_2 b_3 b_4 b_5 b_6 b_7 b_8 b_9 b_10 b_11 b_12 b_13 b_14 b_15
         m1 m2 m3 m4;

tempvar b_1 p1_1 p2_1 p3_1 p4_1 p5_1
            p1_2 p2_2 p3_2 p4_2 p5_2
            p1_3 p2_3 p3_3 p4_3 p5_3
            p1_4 p2_4 p3_4 p4_4 p5_4
            p1_5 p2_5 p3_5 p4_5 p5_5
            p1_6 p2_6 p3_6 p4_6 p5_6
            p1_7 p2_7 p3_7 p4_7 p5_7
            p1_8 p2_8 p3_8 p4_8 p5_8
            p1_9 p2_9 p3_9 p4_9 p5_9
            p1_10 p2_10 p3_10 p4_10 p5_10
            p1_11 p2_11 p3_11 p4_11 p5_11
            p1_12 p2_12 p3_12 p4_12 p5_12
            p1_13 p2_13 p3_13 p4_13 p5_13
            p1_14 p2_14 p3_14 p4_14 p5_14
            p1_15 p2_15 p3_15 p4_15 p5_15

            p1 p2 p3 p4 p5;

quietly { ;
 gen double `p1_1'=0; gen double `p2_1'=0; gen double `p3_1'=0; gen double `p4_1'=0; gen double `p5_1'=0;
 gen double `p1_2'=0; gen double `p2_2'=0; gen double `p3_2'=0; gen double `p4_2'=0; gen double `p5_2'=0;
 gen double `p1_3'=0; gen double `p2_3'=0; gen double `p3_3'=0; gen double `p4_3'=0; gen double `p5_3'=0;
 gen double `p1_4'=0; gen double `p2_4'=0; gen double `p3_4'=0; gen double `p4_4'=0; gen double `p5_4'=0;
 gen double `p1_5'=0; gen double `p2_5'=0; gen double `p3_5'=0; gen double `p4_5'=0; gen double `p5_5'=0;
 gen double `p1_6'=0; gen double `p2_6'=0; gen double `p3_6'=0; gen double `p4_6'=0; gen double `p5_6'=0;
 gen double `p1_7'=0; gen double `p2_7'=0; gen double `p3_7'=0; gen double `p4_7'=0; gen double `p5_7'=0;
 gen double `p1_8'=0; gen double `p2_8'=0; gen double `p3_8'=0; gen double `p4_8'=0; gen double `p5_8'=0;
 gen double `p1_9'=0; gen double `p2_9'=0; gen double `p3_9'=0; gen double `p4_9'=0; gen double `p5_9'=0;
 gen double `p1_10'=0; gen double `p2_10'=0; gen double `p3_10'=0; gen double `p4_10'=0; gen double `p5_10'=0;
 gen double `p1_11'=0; gen double `p2_11'=0; gen double `p3_11'=0; gen double `p4_11'=0; gen double `p5_11'=0;
 gen double `p1_12'=0; gen double `p2_12'=0; gen double `p3_12'=0; gen double `p4_12'=0; gen double `p5_12'=0;
 gen double `p1_13'=0; gen double `p2_13'=0; gen double `p3_13'=0; gen double `p4_13'=0; gen double `p5_13'=0;
 gen double `p1_14'=0; gen double `p2_14'=0; gen double `p3_14'=0; gen double `p4_14'=0; gen double `p5_14'=0;
 gen double `p1_15'=0; gen double `p2_15'=0; gen double `p3_15'=0; gen double `p4_15'=0; gen double `p5_15'=0;

 gen double `p1' = 0; gen double `p2' = 0; gen double `p3' = 0; gen double `p4' = 0; gen double `p5' = 0;
 gen double `b_1'= 0;

   replace `p1_1' = normal(`m1'-`b_1');
   replace `p2_1' = normal(`m2'-`b_1') - normal(`m1'-`b_1');
   replace `p3_1' = normal(`m3'-`b_1') - normal(`m2'-`b_1');
   replace `p4_1' = normal(`m4'-`b_1') - normal(`m3'-`b_1');
   replace `p5_1' = 1 - normal(`m4'-`b_1');
```

```
54
55      replace `p1_2' = normal(`m1'-`b_2');
56      replace `p2_2' = normal(`m2'-`b_2') - normal(`m1'-`b_2');
57      replace `p3_2' = normal(`m3'-`b_2') - normal(`m2'-`b_2');
58      replace `p4_2' = normal(`m4'-`b_2') - normal(`m3'-`b_2');
59      replace `p5_2' = 1 - normal(`m4'-`b_2');
60
61      replace `p1_3' = normal(`m1'-`b_3');
62      replace `p2_3' = normal(`m2'-`b_3') - normal(`m1'-`b_3');
63      replace `p3_3' = normal(`m3'-`b_3') - normal(`m2'-`b_3');
64      replace `p4_3' = normal(`m4'-`b_3') - normal(`m3'-`b_3');
65      replace `p5_3' = 1 - normal(`m4'-`b_3');
66
67      replace `p1_4' = normal(`m1'-`b_4');
68      replace `p2_4' = normal(`m2'-`b_4') - normal(`m1'-`b_4');
69      replace `p3_4' = normal(`m3'-`b_4') - normal(`m2'-`b_4');
70      replace `p4_4' = normal(`m4'-`b_4') - normal(`m3'-`b_4');
71      replace `p5_4' = 1 - normal(`m4'-`b_4');
72
73      replace `p1_5' = normal(`m1'-`b_5');
74      replace `p2_5' = normal(`m2'-`b_5') - normal(`m1'-`b_5');
75      replace `p3_5' = normal(`m3'-`b_5') - normal(`m2'-`b_5');
76      replace `p4_5' = normal(`m4'-`b_5') - normal(`m3'-`b_5');
77      replace `p5_5' = 1 - normal(`m4'-`b_5');
78
79      replace `p1_6' = normal(`m1'-`b_6');
80      replace `p2_6' = normal(`m2'-`b_6') - normal(`m1'-`b_6');
81      replace `p3_6' = normal(`m3'-`b_6') - normal(`m2'-`b_6');
82      replace `p4_6' = normal(`m4'-`b_6') - normal(`m3'-`b_6');
83      replace `p5_6' = 1 - normal(`m4'-`b_6');
84
85      replace `p1_7' = normal(`m1'-`b_7');
86      replace `p2_7' = normal(`m2'-`b_7') - normal(`m1'-`b_7');
87      replace `p3_7' = normal(`m3'-`b_7') - normal(`m2'-`b_7');
88      replace `p4_7' = normal(`m4'-`b_7') - normal(`m3'-`b_7');
89      replace `p5_7' = 1 - normal(`m4'-`b_7');
90
91      replace `p1_8' = normal(`m1'-`b_8');
92      replace `p2_8' = normal(`m2'-`b_8') - normal(`m1'-`b_8');
93      replace `p3_8' = normal(`m3'-`b_8') - normal(`m2'-`b_8');
94      replace `p4_8' = normal(`m4'-`b_8') - normal(`m3'-`b_8');
95      replace `p5_8' = 1 - normal(`m4'-`b_8');
96
97      replace `p1_9' = normal(`m1'-`b_9');
98      replace `p2_9' = normal(`m2'-`b_9') - normal(`m1'-`b_9');
99      replace `p3_9' = normal(`m3'-`b_9') - normal(`m2'-`b_9');
100     replace `p4_9' = normal(`m4'-`b_9') - normal(`m3'-`b_9');
101     replace `p5_9' = 1 - normal(`m4'-`b_9');
102
103     replace `p1_10' = normal(`m1'-`b_10');
104     replace `p2_10' = normal(`m2'-`b_10') - normal(`m1'-`b_10');
105     replace `p3_10' = normal(`m3'-`b_10') - normal(`m2'-`b_10');
106     replace `p4_10' = normal(`m4'-`b_10') - normal(`m3'-`b_10');
107     replace `p5_10' = 1 - normal(`m4'-`b_10');
108
109     replace `p1_11' = normal(`m1'-`b_11');
110     replace `p2_11' = normal(`m2'-`b_11') - normal(`m1'-`b_11');
111     replace `p3_11' = normal(`m3'-`b_11') - normal(`m2'-`b_11');
112     replace `p4_11' = normal(`m4'-`b_11') - normal(`m3'-`b_11');
113     replace `p5_11' = 1 - normal(`m4'-`b_11');
114
115     replace `p1_12' = normal(`m1'-`b_12');
116     replace `p2_12' = normal(`m2'-`b_12') - normal(`m1'-`b_12');
117     replace `p3_12' = normal(`m3'-`b_12') - normal(`m2'-`b_12');
118     replace `p4_12' = normal(`m4'-`b_12') - normal(`m3'-`b_12');
119     replace `p5_12' = 1 - normal(`m4'-`b_12');
120
121     replace `p1_13' = normal(`m1'-`b_13');
122     replace `p2_13' = normal(`m2'-`b_13') - normal(`m1'-`b_13');
123     replace `p3_13' = normal(`m3'-`b_13') - normal(`m2'-`b_13');
```

```
124       replace `p4_13' = normal(`m4'-`b_13') - normal(`m3'-`b_13');
125       replace `p5_13' = 1 - normal(`m4'-`b_13');
126
127       replace `p1_14' = normal(`m1'-`b_14');
128       replace `p2_14' = normal(`m2'-`b_14') - normal(`m1'-`b_14');
129       replace `p3_14' = normal(`m3'-`b_14') - normal(`m2'-`b_14');
130       replace `p4_14' = normal(`m4'-`b_14') - normal(`m3'-`b_14');
131       replace `p5_14' = 1 - normal(`m4'-`b_14');
132
133       replace `p1_15' = normal(`m1'-`b_15');
134       replace `p2_15' = normal(`m2'-`b_15') - normal(`m1'-`b_15');
135       replace `p3_15' = normal(`m3'-`b_15') - normal(`m2'-`b_15');
136       replace `p4_15' = normal(`m4'-`b_15') - normal(`m3'-`b_15');
137       replace `p5_15' = 1 - normal(`m4'-`b_15');
138
139       replace `p1' = normal((`m1'-`b')/`s');
140       replace `p2' = normal((`m2'-`b')/`s') - normal((`m1'-`b')/`s');
141       replace `p3' = normal((`m3'-`b')/`s') - normal((`m2'-`b')/`s');
142       replace `p4' = normal((`m4'-`b')/`s') - normal((`m3'-`b')/`s');
143       replace `p5' = 1 - normal((`m4'-`b')/`s');
144
145       replace `lnf' = (vig1==1)*ln(`p1_1')+(vig1==2)*ln(`p2_1')+(vig1==3)*ln(`p3_1')+(vig1==4)*
      ln(`p4_1')+(vig1==5)*ln(`p5_1')
146                       +(vig2==1)*ln(`p1_2')+(vig2==2)*ln(`p2_2')+(vig2==3)*ln(`p3_2')+(vig2==4)*
      ln(`p4_2')+(vig2==5)*ln(`p5_2')
147                       +(vig3==1)*ln(`p1_3')+(vig3==2)*ln(`p2_3')+(vig3==3)*ln(`p3_3')+(vig3==4)*
      ln(`p4_3')+(vig3==5)*ln(`p5_3')
148                       +(vig4==1)*ln(`p1_4')+(vig4==2)*ln(`p2_4')+(vig4==3)*ln(`p3_4')+(vig4==4)*
      ln(`p4_4')+(vig4==5)*ln(`p5_4')
149                       +(vig5==1)*ln(`p1_5')+(vig5==2)*ln(`p2_5')+(vig5==3)*ln(`p3_5')+(vig5==4)*
      ln(`p4_5')+(vig5==5)*ln(`p5_5')
150                       +(vig6==1)*ln(`p1_6')+(vig6==2)*ln(`p2_6')+(vig6==3)*ln(`p3_6')+(vig6==4)*
      ln(`p4_6')+(vig6==5)*ln(`p5_6')
151                       +(vig7==1)*ln(`p1_7')+(vig7==2)*ln(`p2_7')+(vig7==3)*ln(`p3_7')+(vig7==4)*
      ln(`p4_7')+(vig7==5)*ln(`p5_7')
152                       +(vig8==1)*ln(`p1_8')+(vig8==2)*ln(`p2_8')+(vig8==3)*ln(`p3_8')+(vig8==4)*
      ln(`p4_8')+(vig8==5)*ln(`p5_8')
153                       +(vig9==1)*ln(`p1_9')+(vig9==2)*ln(`p2_9')+(vig9==3)*ln(`p3_9')+(vig9==4)*
      ln(`p4_9')+(vig9==5)*ln(`p5_9')
154                       +(vig10==1)*ln(`p1_10')+(vig10==2)*ln(`p2_10')+(vig10==3)*ln(`p3_10')+(
      vig10==4)*ln(`p4_10')+(vig10==5)*ln(`p5_10')
155                       +(vig11==1)*ln(`p1_11')+(vig11==2)*ln(`p2_11')+(vig11==3)*ln(`p3_11')+(
      vig11==4)*ln(`p4_11')+(vig11==5)*ln(`p5_11')
156                       +(vig12==1)*ln(`p1_12')+(vig12==2)*ln(`p2_12')+(vig12==3)*ln(`p3_12')+(
      vig12==4)*ln(`p4_12')+(vig12==5)*ln(`p5_12')
157                       +(vig13==1)*ln(`p1_13')+(vig13==2)*ln(`p2_13')+(vig13==3)*ln(`p3_13')+(
      vig13==4)*ln(`p4_13')+(vig13==5)*ln(`p5_13')
158                       +(vig14==1)*ln(`p1_14')+(vig14==2)*ln(`p2_14')+(vig14==3)*ln(`p3_14')+(
      vig14==4)*ln(`p4_14')+(vig14==5)*ln(`p5_14')
159                       +(vig15==1)*ln(`p1_15')+(vig15==2)*ln(`p2_15')+(vig15==3)*ln(`p3_15')+(
      vig15==4)*ln(`p4_15')+(vig15==5)*ln(`p5_15')
160
161                       +(y==1)*ln(`p1')+(y==2)*ln(`p2')+(y==3)*ln(`p3')+(y==4)*ln(`p4')+(y==5)*ln
      (`p5');
162
163     };
164   end;
165   #delimit cr
166
167
168
169
170
```
